# Ra-223 induces clustered DNA damage and inhibits cell survival in several prostate cancer cell lines

**DOI:** 10.1016/j.tranon.2022.101543

**Published:** 2022-09-18

**Authors:** Andris Abramenkovs, Mehran Hariri, Diana Spiegelberg, Sten Nilsson, Bo Stenerlöw

**Affiliations:** a Department of Immunology, Genetics and Pathology, Rudbeck Laboratory, Uppsala University, Uppsala SE-75185, Sweden; b Department of Surgical Sciences, Uppsala University, Uppsala, Sweden; c Department of Oncology-Pathology, Karolinska Institute, Stockholm, Sweden

**Keywords:** Prostate cancer, DNA damage, Ra-223, α-particle, HAp

## Abstract

The bone-seeking radiopharmaceutical Xofigo (Radium-223 dichloride) has demonstrated both extended survival and palliative effects in treatment of bone metastases in prostate cancer. The alpha-particle emitter Ra-223, targets regions undergoing active bone remodeling and strongly binds to bone hydroxyapatite (HAp). However, the toxicity mechanism and properties of Ra-223 binding to hydroxyapatite are not fully understood. By exposing 2D and 3D (spheroid) prostate cancer cell models to free and HAp-bound Ra-223 we here studied cell toxicity, apoptosis and formation and repair of DNA double-strand breaks (DSBs). The rapid binding with a high affinity of Ra-223 to bone-like HAp structures was evident (KD= 19.2 × 10^−18^ M) and almost no dissociation was detected within 24 h. Importantly, there was no significant uptake of Ra-223 in cells. The Ra-223 alpha-particle decay produced track-like distributions of the DNA damage response proteins 53BP1 and ɣH2AX induced high amounts of clustered DSBs in prostate cancer cells and activated DSB repair through non-homologous end-joining (NHEJ). Ra-223 inhibited growth of prostate cancer cells, independent of cell type, and induced high levels of apoptosis. In summary, we suggest the high cell killing efficacy of the Ra-223 was attributed to the clustered DNA damaged sites induced by α-particles.

## Highlights

• Ra-223 binding to bone-like structures is extreme without any uptake in cancer cells.
• Clustered DNA double-strand break (DSB) were induced in prostate tumor cells.
• These DSB activated non-homologous end-joining (NHEJ) repair.
• This triggered apoptosis and inhibited cell growth independent of cell type.
• Ra-223 bound to bone-like structures increases the tumor cell-killing.

## Introduction

Targeted alpha therapy (TAT) using radium-223 is one of several treatment options for metastatic castration-resistant prostate cancer (CRPC). Ra-223 dichloride is a calcium analog that binds to hydroxyapatite (HAp) which is a natural bone mineral [1,2]. As the Ra-223 quickly localizes to the bone structures in the body [3], it is applicable for patients with metastases only found in bone. The metastatic CRPC patient undergoing Ra-223 treatment show an extended median survival of 3.6 month and improved life quality due to reduced pain [4] The Ra-223 is an α-emitter with half-life of 11.4 days and the high-linear energy transfer (high-LET) α-particles originating from the decay chain have a range of approximately 70 µm in tissue [5]. In general, DSBs induced by high-LET irradiation are complex and difficult to repair [6], produce chromosomal aberrations and eventually induce cell death. The cell killing effect of high-LET radiation is almost independent of the oxygen level and cell cycle stage which is a significant challenge for conventional radiotherapy [7]. Despite the above evidence of high-LET radiation effect, the literature on basic properties and radiobiological effects of Ra-223 are scarce, including data on the formation of clustered DSB, activation of DNA repair and mechanisms of tumor cell killing. Such information could be important for optimization of therapies combining Ra-223 and drugs.

In this study, we show that free and HAp-bound Ra-223 treatment reduced cell survival independently of prostate cancer cell type. Furthermore, Ra-223 induced high levels of apoptosis and was able to inhibit the growth of 3D-spheroids. The distribution and repair of DSBs after exposure to the Ra-223 was consistent with a high-LET irradiation. We conclude that the DNA damage induced by the α-particles from Ra-223 decay chain is the major contributor to the cell killing effect.

## Methods

### Cell culture

Prostate cancer PC3 and brain metastatic prostate cancer DU145 cells were kindly provided by Prof. Anna Orlova, Uppsala University, Sweden. Prostate cancer 22RV1 cells were purchased from American Type Culture Collection (Manassas, VA, USA). Cells were grown in RPMI containing 9% FBS (Sigma Aldrich, MO, USA), 100 IU/ml penicillin and streptomycin and 2 mM l-glutamine (Biochrom Kg, Berlin, Germany) and maintained at 37 °C in 5% CO_2_.

### Surface functionalization with hydroxyapatite

Plastic 6-well and 12-well plates (Thermo Fisher Scientific, Sweden) were functionalized by HAp according to the described procedure [8]. In brief, plates were incubated with 0.5 mg/ml HSA (Sigma-Aldrich) in 1X PBS for more than 3 h. Then 100 mM of CaCl_2_ (Merck) in 50% ethanol was added for 2 min, washed twice in 50% ethanol and incubated for 2 min with 100 mM K_2_HPO_4_ (Merck) in 50% ethanol. Then plates were incubated in simulated body fluid for 24 h at 36.5ºC before washing with a MQ water and air drying on the next day. The plates were sterilized under UV light before use*.* The surface uniformity varied over the HAp plates (typically +/−25%) and the exact distribution of Ra-223 in the HAp layers was not investigated.

### Clonogenic assay

#### Free Ra-233

Cells (PC3, 22RV1, DU145) were pre-plated in 6 well plates (100–200 cells for control and treatment) one day before the cell medium was replaced with 2 ml of Ra-223 medium (0–500 Bq/ml) and incubated for 8–14 days at 37ºC. Cell were fixed and stained with crystal violet (Sigma Aldrich). Colonies containing ˃50 cells were scored and the survival was estimated as a ratio of the treatment group colony number versus control.

### HAp

The HAp coated 6 well plates, were pre-treated with 2 ml medium containing 0 or 500 Bq/ml Ra-223 for 2 h at 37ºC, and unbound Ra-223 was removed. Then cells (PC3, 22RV1) were seeded (control: 400 and treatment: 8000 cells) on the radioactive HAp surface and incubated with 2 ml of non-radioactive medium containing 9% FBS to grow at 37ºC for 8–14 days. The cells fixed, stained, and counted as described above.

### Spheroid assays

#### Spheroid assays with free Ra-223

Spheroids of 22RV1 were produced by plating 1000 cells in the ultra-low attachment 96 well plates (Corning) and incubated for 2–3 days before adding 200 µL of 0- 500 Bq/ml Ra-223 medium. Images were acquired every two days until day 18 and spheroid volumes were scored using a custom Fiji macro [9], assuming spherical geometries.

#### Spheroid outgrowth assay on HAp coated plates

Spheroids from 22RV1 and DU145 cells were produced by plating 1000 and 500 cells, respectively, as described above. Spheroids were transferred into HAp precoated 12-well plates to adhere for 24 h. Then growth medium was exchanged with 2 ml of 0, 500 or 1000 Bq/ml Ra-223 medium and incubated for two hours before replacing it with non-radioactive media. Images were acquired every two days until day 10 and the areas were evaluated manually using Fiji. The outgrowing area was normalized to the corresponding area at day 0.

### Apoptosis

Cells (PC3, 22RV1, DU145) were plated in T25 flasks (VWR) 24 h before incubation with or without 2 ml of 1 kBq/ml of Ra-223 medium for 48 h. After the removal of Ra-223, cells were incubated with a non-radioactive medium at 37 °C for 24 h. Apoptosis assay was performed using Alexa Fluor ^R^ ©488 Annexin V/Dead Cell Apoptosis Kit with Alexa Fluor 488 annexin V and PI for flow cytometry and CellEvent™ Caspase-3/7 Green Flow Cytometry Assay Kit (Thermo Fisher Scientific) as previously described [10].

### DSB detection by pulsed-field gel electrophoresis (PFGE)

DU145 cells were labeled with 2000 Bq/ml [methyl-C-14]thymidine (Perkin Elmer) in a complete medium and embedded in 100 µl agarose plugs at 37 °C (0.6% w/v, Sigma-Aldrich) at a concentration of 2 × 10 [6] cells per ml. I.) X-rays: After gelling for 20 min at 0 °C, plugs with cells were transferred to tubes and irradiated on ice with 100 Gy of 6 MV X-rays (Elekta Precise Treatment System, Uppsala University Hospital) at a dose rate of 4.5 Gy/min. II.) Ra-223: cells were incubated in ∼ 1 MBq/ml Ra-223 solution for 6 h on ice on an orbital shaker, followed by 3 washes in 1X PBS and embedded in agarose. After X-ray or Ra-223 exposure, cells were prepared for PFGE, and DNA fragments were analyzed as described [8,11].

### Immunofluorescence and image acquisition of repair foci

Cells were plated in 4-well chamber slides (Nunc) were cooled on ice for 20 min before incubating with ice-cold 1 ml of 100 kBq/ml Ra-223 for 1 h. After removing Ra-223 media, cells were washed three times with ice-cold media. The cells were let repair for indicated time (0, 60, 240, 1440 min) at 37ºC. Cells were then washed in 1X PBS and fixated in 99% methanol at −20ºC. Staining with antibodies against ɣH2AX (EMD Millipore Merck Darmstadt, Germany) and 53BP1 (Abcam, Cambridge, United Kingdom), image acquisition and analysis were performed as described [10]. The volume rendering was performed in the Imaris software (v 9.2, Bitplane AG).

### DSB repair evaluation by flow cytometry

Cells seeded in 6-well plates were pre-cooled on ice for 20 min followed by adding 2 ml of ice-cold medium containing 300 kBq/ml Ra-223. After 1 h on ice to block DSB repair, the radioactive medium was removed and the cells were washed three times with an ice-cold medium. Then warm fresh medium were added to cells for repair in different time points (0, 15, 30, 60, 240 min). DSB repair was analyzed by measuring phosphorylation of DNA-PKcs (T2609) (Abcam, ab18356, 10B) as described before [12].

### Western blot

Whole-cell lysates of cells were prepared as described [10]. Briefly, samples were separated using SDS PAGE and transferred to a nitrocellulose membrane (Immobilon-P Transfer membrane, Millipore, Merck) by wet blotting. The membrane was blocked for 1 h in PBS with 5% BSA and incubated with a monoclonal anti-Arv7 antibody (1:1000; #19,672, E3O8L, Cell signaling, Beverly, MA, USA) and an anti-beta actin antibody (1:10,000) (Sigma-Aldrich), at 4 °C overnight. After several washing steps, a species-specific Horse Radish Peroxidase-labelled secondary antibody was applied for 1 h at the room temperature. Immunoreactive bands were visualized with a CCD camera (SuperCCD HR, Fujifilm) after treatment with electrochemiluminescent solution (Immobilon, Millipore).

### Real-time affinity measurements

The HAp coated surface real-time affinity with Ra-223 and DU145 cells uptake and retention of activity was measure with LigandTracer technology as described [13]. In brief Petri dishes (10 cm diameter) were tilted to coat 1/8 of surface area with HAp for measuring Ra-223 binding using LigandTracer (LigandTracer White, Ridgeview AB). After background measurements, 2 ml of 5 kBq/ml Ra-223 was added to the petri dish and the association was measured for 2 h. Then the radioactive medium was replaced with 3 ml of non-radioactive media to measure dissociation overnight. The affinity was estimated with the TraceDrawer software (Ridgeview AB) using one to one binding model with a constant dissociation constant and data acquired within 3 h after the start of dissociation.

In the live-cell experiments, 1 million DU145 cells were plated on inclined dishes one day before the experiments. The LigandTracer instrument was equilibrated in an incubator at 37 °C in 5% CO2 for at least 5 h before measurements. The dish was placed in the instrument and the association was measured for 4 h when 2 ml of 5 kBq/ml Ra-223 medium was added to the cells. The dissociation was measured overnight when the cell medium was replaced with new non-radioactive media.

### Statistical analysis

Statistical analysis was performed in GraphPad Prism v 6.07 (La Jolla, CA, USA). *p*-values <0.05 were considered statistically significant. Multiple comparisons were performed using a two-way ANOVA with Bonferroni correction.

## Results

### Cell survival using free Ra-233

**Fig. 1:**
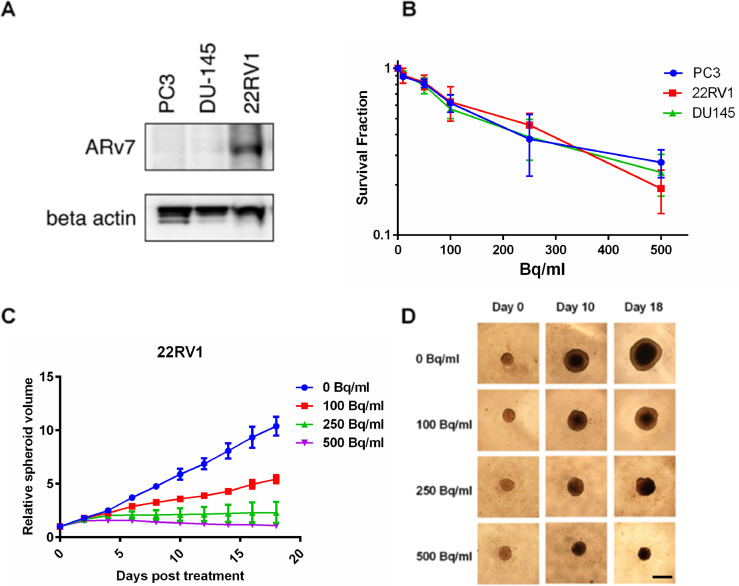
Ra-223 reduces the cell survival independently of ARv7 status. (A) The expression of ARv7 in the PC3, DU145 and 22RV1 cells. Representative image from 3 Western blot measurements. (B) The DU145, PC3 and 22RV1 cell colony forming ability is reduced when exposed to Ra-223. Cells were treated with 0- 500 Bq/ml Ra-223 and colonies with ˃50 cells were scored. Mean values with SEM from 3 to 4 independent experiments are shown. (C) 22RV1 spheroids were treated with 0- 500 Bq/ml Ra-223 and their volume was measured every 2 days. Mean values with SEM from 3 independent experiments are shown. (D) Representative images of 22RV1 spheroids at day 0, 10 and 18 with 500 µm scale bar.

The survival of all three cell lines significantly decreased after increasing the activity concentration of free Ra-223 treatment. All cell lines showed similar radiosensitivity in this assay (Fig. 1B) comparable with our previous results for high-LET irradiation [14]. Expression of ARv7 in prostate cancer cells is associated with ADT resistance and decreased survival of patients [14]. Western blot analysis confirmed ARv7 expression in 22RV1 cells while PC3 and DU145 cells had no detectable ARv7 levels (Fig. 1A) and the results indicates that the expression of ARv7 did not affect the survival of cells when exposed to the Ra-223 (Fig. 1B).

### Cell survival using HAp-coated plates

The survival of PC3 and 22RV1 cells on the Ra-223 pre-treated HAp surface (500 Bq/ml) showed a drastic reduction (100-fold) compare to free Ra-223 treatment at the same administered activity (Fig. 5C and D).

### Spheroid growth

22RV1 spheroid growth was significantly reduced after 6 days of exposure to 250 Bq/ml (*p* = 0.0248) and 500 Bq/ml free Ra-223 (*p* = 0.0019). Treatment with 100 Bq/ml Ra-223, showed significantly reduced growth (*p* = 0.0182) after 8 days (Fig. 1C). At the end of treatment (18 days), 250 and 500 Bq/ml groups showed complete growth inhibition, and the lowest activity (100 Bq/ml) reduced the growth by 50% compared to control. Representative spheroid images are shown in Fig. 1D. PC3 cells did not form spheroids and untreated DU145 spheroids lost viability over time (data not shown).

To mimic *in vivo* conditions, we tested how spheroid's outgrowth was affected when plated on a HAp surface pre-treated with or without Ra-223 (Fig. 5A, B, and Suppl. Fig. 3). 22RV1 spheroid outgrowth was significantly reduced by day 4 in 0.5 kBq/ml and 1 kBq/ml groups (Fig. 5A; *p* < 0.04) with persistent effect until day 10 (end of the experiment). DU145 spheroid outgrowth was not affected by Ra-223 treatment (Suppl. Fig. 3). Yet, the treatment reduced the density of the cells in the center and periphery, thus rendering the scoring of cellular outgrowth difficult at later stages.

### Apoptosis induction

**Fig. 2:**
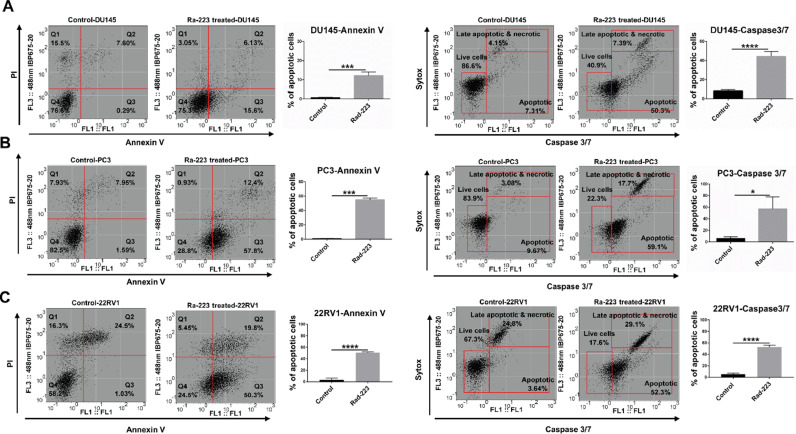
Ra-223 induce high levels of apoptosis in prostate cancer cells. Annexin V/PI and Caspase 3/7-sytox flow cytometric analysis of apoptosis after 48 h incubation with 1000 Bq/ml Ra-223 followed by 24 h incubation without Ra-223. (A) DU145, (B) PC3, and (C) 22RV1 cells. The lower right quarter shows apoptotic cell's percentage. Live cells are captured in the lower-left square. Means and SE from three independent experiments are shown. Student *t*-test was used to analyze the statistical differences between samples with * *p* < 0.05, ** *p* < 0.01, *** *p* < 0.001, and **** *p* < 0.0001.

Cells were treated with 1000 Bq/ml Ra-223 for 48 h to quantify cell apoptosis by flow cytometric analysis of caspase 3/7-sytox and Annexin V/PI double staining. The representative graphs and data analysis in three cell lines are shown in Fig. 2. After exposure to Ra-223, more than 55% of the PC3 and 22RV1 cells were apoptotic as detected by Annexin V, whereas fewer DU145 cells were positive for Annexin V. These initial results were confirmed by analysis of Caspase 3/7 (Fig. 2, Suppl. Fig. 1)

### DSB detection (PFGE)

**Fig. 3:**
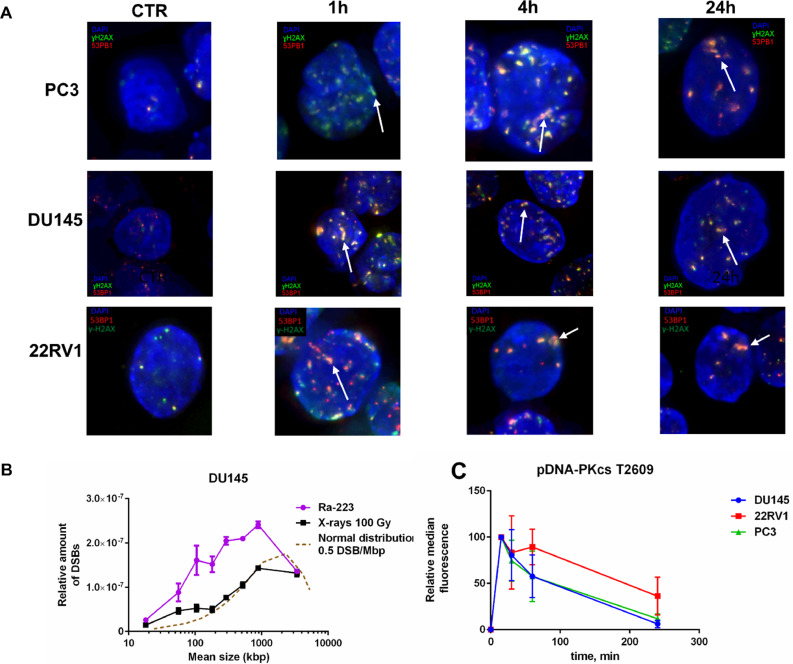
Ra-223 induces DNA damage consistent with high-LET irradiation. (A) Ra-223 exposure induces track-like 53BP1 (red) and ɣH2AX (green) foci distribution (white arrows). PC3, DU145, and 22RV1 cells were treated for 1 h on ice with 100 kBq/ml Ra-223 and allowed to repair for indicated time. Representative images from 3 independent experiments are shown. (B) Ra-223 induces DNA fragmentation and accumulation of small DNA fragments. DU145 cells were exposed to ∼1MBq/ml of Ra-223 for 6 h (purple line) or irradiated with 100 Gy of X-rays (black line) on ice and distribution of DNA fragments were analyzed. In comparison, the normal distribution of 0.5 DSB/ Mbp is shown (brown, dashed line). The data represents the mean values from two independent experiments and error bars correspond to SEM. (C) Ra-223 induced damage activates NHEJ repair. The PC3 (green line), DU145 (blue line), and 22RV1 cells (red line) were exposed to 300 kBq/ml Ra-223 for 1 h and the pDNA-PKcs T2609 levels were assessed by flow cytometry at 15- 240 min post irradiation. Data from 3 independent experiments are shown and mean values with SEM are displayed.

The DU145 cell's DNA fragmentation pattern was analyzed by irradiation on ice using PFGE to test the consistency of Ra-233 with high-LET radiation DNA damage (Fig. 3B). DSB distribution was random for X-ray and non-random for Ra-223 with a high excess of small DNA fragments (<1 Mbp sizes). The induction of an excess of relatively small DNA fragment (<1 Mbp) is a hallmark of high-LET radiation, but because of the low frequency of these fragments, high doses are needed. Cells were here irradiated at 0 °C to avoid any repair of DSB.

### DNA damage foci formation

Unrepaired or misrepaired DSBs are the major cause of radiation-induced cell death. The ɣH2AX and 53BP1 foci were analyzed as DNA damage and repair biomarkers (Fig. 3A). Structured-illumination microscopy and volume rendering showed that α-particles induced track-like distribution of 53BP1 foci (Supp. Fig. 2).

### Flow cytometry analysis of DSB repair

DNA-PKcs phosphorylation at T2609 was analyzed by flow cytometry to score DSBs repair. PC3 and DU145 cells quickly repaired DSBs and in 4 h post-irradiation only ∼ 6–11% of the initial DSBs were left unrepaired, whereas repair in 22RV1 cells was less evident (Fig. 3C).

### Real-time affinity measurements of HAp and DU145 cells with Ra-223

**Fig. 4:**
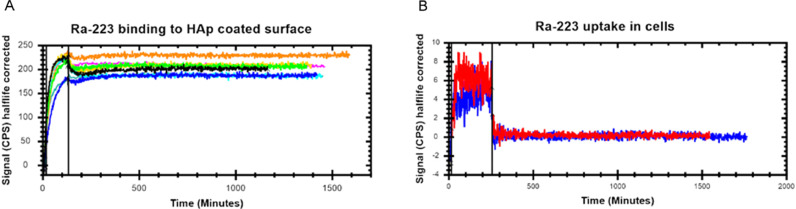
The Ra-223 binds to HAp without being taken up in cells. (A) The kinetics of the Ra-223 binding to the HAp-coated surface. Data from 8 independent experiments are shown in the different colors. (B) The Ra-223 is not taken up in cells. Data from 2 independent experiments are shown in the different colors. The solid vertical lines separate association from the dissociation.

Ra-223 binds strongly to active bone remodeling sites [5].To characterize binding, a part of a petri dish coated with HAp and Ra-223 binding was quantified (Fig. 4A). Ra-223 bound quickly to the HAp-coated surface with >99% of maximum signal achievement within 2 h. After removal of Ra-223, almost no dissociation was detected within the following 24 h. The affinity was estimated to be 19.2 ± 6.5 × 10^−18^ M (mean ± SD; *n* = 9). The small fraction of Ra-223 associated with the surface of cells quickly dissociated when a non-radioactive medium was introduced, indicating that no Ra-223 was taken up in cells within 2 h (Fig. 4B).

**Fig. 5:**
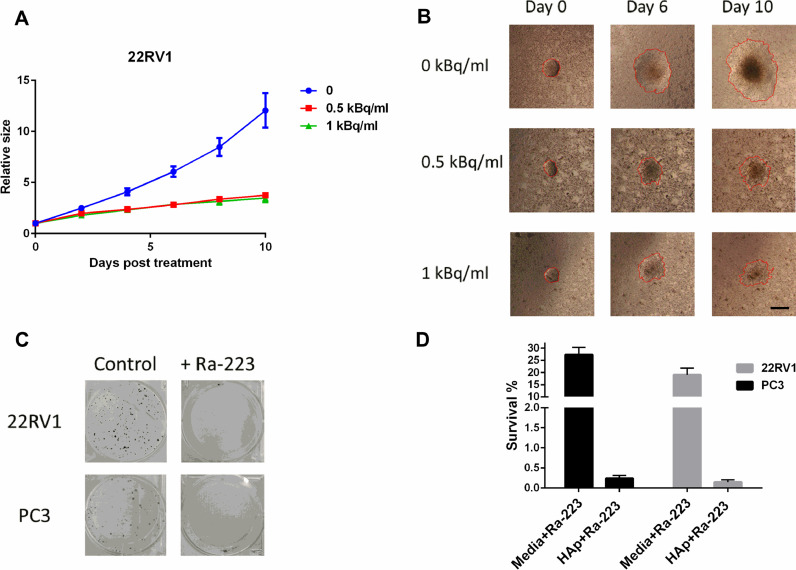
HAp surface pre-treated with Ra-223 significantly reduces the survival and outgrowth of cells. HAp surface were pre-incubated with Ra-223 (50–1000 Bq/ml, 2 h), washed followed by seeding of spheroids (A, B) and cells (C). (A) HAp surface treatment with Ra-223 decreases the 22RV1 spheroid outgrowth on surface. Data represent mean values from 3 independent experiments and error bars correspond to the SD. (B) Representative images of the 22RV1 spheroid outgrowth on day 0, 6 and 10 (scale bar = 500 µm). Red line shows the scored area and the boarder of outgrowing cells. (C) The Ra-223 pretreated HAp surface reduces the cell survival. Representative images from 3 independent experiments are shown. (D) Survival of PC3 and 22RV1 cells from 3 independent experiments are shown for HAp-coated surface (500 Bq/ml, 2 h) and compared to the Ra-223 treatment in media (500 Bq/ml data from Fig. 1 B).

## Discussion

The DNA double-strand break (DSB) is considered as the most critical DNA damage induced by ionizing radiation and unrepaired or misrepaired DSBs may lead to cell death or changes in the genome stability. Although quantification of DNA damage induced by Ra-223 has been attempted [15], the use of conventional DNA damage foci counting methods is questionable as several DSBs induced by the α-particles are distributed in close proximity and thus one focus could represent many DSBs [16]. By using PFGE to analyze DNA fragmentation distributions, we here show that Ra-223 induces clustered DSBs and an excess of small DSBs fragments (<1 Mbp sizes) in prostate cancer cells, consistent with studies measuring DNA damage after high-LET ion irradiation [11,17]. Such clustering is partly due to the increased probability of the high-LET particles producing track-like, multiple DSBs, when intersecting folded chromatin structures in the cells [18]. These data were also supported by the track-like distributions of ɣH2AX and 53BP1 foci. As scoring of DNA repair after high-LET irradiation is not possible using standard foci scoring assay and analysis with PFGE has difficulties to detect very small fragments which are produced in excess after the high-LET irradiation, we scored DSB repair using the relative fluorescence intensity of phosphorylated (T2609) DNA-PKcs, a key protein in DSB repair by non-homologous end-joining (NHEJ), as a marker of the DSBs [12]. Clearly, these clustered DSB activated NHEJ repair and the early repair kinetics measured by flow cytometry was comparable to results from previous PFGE analysis measuring rejoining of DNA after ion irradiations [19], suggesting that the high toxicity of the DNA damage prompted by the high-LET irradiation stems from the clustering of highly complex DSBs.

In this study, we used three prostate cancer cell lines, including the ARv7 expressing 22RV1, with different sensitivity to low-LET radiation [20]. We show that Ra-223 reduced cell survival independently of the cell type, suggesting the potential use of Ra-223 treatment in bone metastases exhibiting ADT resistance. Exposure to Ra-223 distributed in the growth media was able to decrease the cell survival in monolayers and 3D spheroids and induce high levels of apoptosis, even though, α-particles from Ra-223 decay have a maximum range of 70 µm in tissue [5]. However, in the experimental settings where Ra-223 is distributed evenly in the media, only a small fraction of the α-particles will hit the cells, which does not reflect the actual situation *in vivo*. To address this issue we quantified Ra-223 binding to HAp surface and results were consistent with previous observations that Ra-223, when injected into the bloodstream, is redistributed to the bones within 2 h [21]. Ra-223 is not taken up in cells, indicating that all damages are induced by decay events outside the cell. Cells and spheroids deposited on a HAp surface with bound Ra-223 displayed reduced cell growth compared to exposure to Ra-223 in the media. However, whereas the outgrowth of 22RV1 spheroids was dramatically reduced, the effect on DU145 spheroids was less evident, indicating that Ra-233 may not be able to inhibit cell migration in all cases. Independently of the cellular outgrowth characteristics, it is possible that at early stages, upper layers of the 3D cell structure are not damaged due to the limited range of α-particles, rendering the treatment less efficacious in larger lesions. Alternatively, it is possible to speculate that cells undergo apoptosis at later stages, several days post-treatment. The HAp surface loaded with Ra-223 decreased cell survival more than unbound Ra-223 in monolayers, possibly further reducing the ability of the circulating tumor cells to attach and form new metastatic sites in the bones and illustrating the efficacy of Ra-223 to reduce the growth of single cells and smaller lesions. However, a direct comparison of results from the different exposure conditions needs proper dose estimates, taking all Ra-223 decays into account (i.e. alpha, beta and gamma). The absence of cellular uptake of Ra-223 and the several-fold higher cell toxicity when Ra-223 is enriched on dish surface, suggests that the majority of the absorbed dose *in vivo* is deposited around the bones, which is consistent with biodistribution measurements [21,22]. The biological effects of alpha-particles are relatively insensitive to modifications of the radiation-response seen after low-LET X-ray exposure. However, recent studies indicate that patients resistant to PSMA-targeted alpha-radiation often harbor DNA damage repair-related gene mutations [23] and that anti-beta 1 integrin targeting can enhance Ra-223 treatment outcomes in pre-clinical models [24]. Although such effects could be due to selection bias or tissue targeting properties, recent findings suggesting indirect bystander effects [25] highlight the complexity of the biological response to these treatments.

Taken together, our data indicate that the DSBs induced by high-LET irradiation can be repaired, yet the repair of highly complex DNA damage results in errors. These errors subsequently lead to chromosomal abnormalities which eventually induce mitotic catastrophe, apoptosis, or senescence. The high levels of apoptosis in all three cell lines are in line with previous investigations [26] correlating high-LET induced clustered DNA damage with an early onset of apoptosis.

ARv7 expression in circulating prostate tumor cells, giving rise to metastases, leads to disease progression and ADT response reduction [27]. Our results demonstrated that cell survival of three cell lines with and without ARv7 expression was decreased after Ra-223 treatment, but further studies are needed to investigate the role of ARv7 expression in the response to α-particles. Furthermore, it is worthwhile to consider in future studies of Ra-223 direct media exposure, to add beta and gamma irradiation control to be able to refine the net effect of alpha particles attenuation in solution.

## CRediT authorship contribution statement

**Andris Abramenkovs:** Conceptualization, Visualization, Data curation, Formal analysis, Investigation, Writing – original draft, Writing – review & editing. **Mehran Hariri:** Data curation, Formal analysis, Investigation, Writing – original draft, Writing – review & editing. **Diana Spiegelberg:** Data curation, Formal analysis, Investigation, Writing – original draft. **Sten Nilsson:** Conceptualization, Visualization, Writing – review & editing. **Bo Stenerlöw:** Conceptualization, Visualization, Data curation, Formal analysis, Investigation, Writing – original draft, Writing – review & editing, Funding acquisition, Supervision.

## Declaration of Competing Interest

None.

## Appendix Supplementary materials

Image, application 1

## References

[1] Parker C., Heidenreich A., Nilsson S., Shore N. (2018). Current approaches to incorporation of radium-223 in clinical practice. Prostate Cancer Prostatic Dis..

[2] Poeppel T.D., Handkiewicz-Junak D., Andreeff M. (2018). EANM guideline for radionuclide therapy with radium-223 of metastatic castration-resistant prostate cancer. Eur. J. Nucl. Med. Mol. Imaging.

[3] Yoshida K., Kaneta T., Takano S. (2016). Pharmacokinetics of single dose radium-223 dichloride (BAY 88-8223) in Japanese patients with castration-resistant prostate cancer and bone metastases. Ann. Nucl. Med..

[4] Parker C., Nilsson S., Heinrich D. (2013). Alpha emitter radium-223 and survival in metastatic prostate cancer. N. Engl. J. Med..

[5] Abou D.S., Ulmert D., Doucet M., Hobbs R.F., Riddle R.C., Thorek D.L. (2016). Whole-body and microenvironmental localization of radium-223 in naive and mouse models of prostate cancer metastasis. J. Natl. Cancer Inst..

[6] Carter R.J., Nickson C.M., Thompson J.M., Kacperek A., Hill M.A., Parsons J.L. (2018). Complex DNA damage induced by high linear energy transfer alpha-particles and protons triggers a specific cellular DNA damage response. Int. J. Radiat. Oncol. Biol. Phys..

[7] Wenzl T., Wilkens J.J. (2011). Theoretical analysis of the dose dependence of the oxygen enhancement ratio and its relevance for clinical applications. Radiat. Oncol..

[8] Iijima K., Suzuki R., Iizuka A., Ueno-Yokohata H., Kiyokawa N., Hashizume M. (2016). Surface functionalization of tissue culture polystyrene plates with hydroxyapatite under body fluid conditions and its effect on differentiation behaviors of mesenchymal stem cells. Colloids Surf. B.

[9] Spiegelberg D., Dascalu A., Mortensen A.C. (2015). The novel HSP90 inhibitor AT13387 potentiates radiation effects in squamous cell carcinoma and adenocarcinoma cells. Oncotarget.

[10] Mortensen A.C.L., Mohajershojai T., Hariri M., Pettersson M., Spiegelberg D. (2020). Overcoming limitations of cisplatin therapy by additional treatment with the HSP90 inhibitor Onalespib. Front. Oncol..

[11] Hoglund E., Blomquist E., Carlsson J., Stenerlow B. (2000). DNA damage induced by radiation of different linear energy transfer: initial fragmentation. Int. J. Radiat. Biol..

[12] Abramenkovs A., Stenerlow B. (2017). Measurement of DNA-dependent protein kinase phosphorylation using flow cytometry provides a reliable estimate of DNA repair capacity. Radiat. Res..

[13] Björke H., Andersson K. (2006). Automated, high-resolution cellular retention and uptake studies *in vitro*. Appl. Radiat. Isot..

[14] Stenerlöw B., Pettersson O.A., Essand M., Blomquist E., Carlsson J. (1995). Irregular variations in radiation sensitivity when the linear energy transfer is increased. Radiother. Oncol..

[15] Schumann S., Eberlein U., Muhtadi R., Lassmann M., Scherthan H. (2018). DNA damage in leukocytes after internal *ex-vivo* irradiation of blood with the α-emitter Ra-223. Sci. Rep..

[16] Costes S.V., Boissiere A., Ravani S., Romano R., Parvin B., Barcellos-Hoff M.H. (2006). Imaging features that discriminate between foci induced by high- and low-LET radiation in human fibroblasts. Radiat. Res..

[17] Claesson A.K., Stenerlöw B., Jacobsson L., Elmroth K. (2007). Relative biological effectiveness of the α-particle emitter 211At for double-strand break induction in human fibroblasts. Radiat. Res..

[18] Radulescu I., Elmroth K., Stenerlow B. (2004). Chromatin organization contributes to non-randomly distributed double-strand breaks after exposure to high-LET radiation. Radiat. Res..

[19] Gustafsson A.S., Hartman T., Stenerlow B. (2015). Formation and repair of clustered damaged DNA sites in high LET irradiated cells. Int. J. Radiat. Biol..

[20] Fraser M., Zhao H., Luoto K.R. (2012). PTEN deletion in prostate cancer cells does not associate with loss of RAD51 function: implications for radiotherapy and chemotherapy. Clin. Cancer Res..

[21] Yoshida K., Kaneta T., Takano S. (2016). Pharmacokinetics of single dose radium-223 dichloride (BAY 88-8223) in Japanese patients with castration-resistant prostate cancer and bone metastases. Ann. Nucl. Med..

[22] Chittenden S.J., Hindorf C., Parker C.C. (2015). A phase 1, open-label study of the biodistribution, pharmacokinetics, and dosimetry of 223Ra-dichloride in patients with hormone-refractory prostate cancer and skeletal metastases. J. Nucl. Med..

[23] Kratochwil C., Giesel F.L., Heussel C.P. (2020). Patients resistant against PSMA-targeting α-radiation therapy often harbor mutations in DNA damage-repair–associated genes. J. Nucl. Med..

[24] Paindelli C., Casarin S., Wang F. (2021). Enhancing Radium 223 treatment efficacy by anti-beta 1 integrin targeting. J. Nucl. Med..

[25] Leung C.N., Canter B.S., Rajon D. (2020). Dose-dependent growth delay of breast cancer xenografts in the bone marrow of mice treated with 223Ra: the role of bystander effects and their potential for therapy. J. Nucl. Med..

[26] Meijer A.E., Jernberg A.R., Heiden T. (2005). Dose and time dependent apoptotic response in a human melanoma cell line exposed to accelerated boron ions at four different LET. Int. J. Radiat. Biol..

[27] Antonarakis E.S., Lu C., Luber B. (2015). Androgen receptor splice variant 7 and efficacy of taxane chemotherapy in patients with metastatic castration-resistant prostate cancer. JAMA Oncol..

