# Supplementary material for: Ra-223 induces clustered DNA damage and inhibits cell survival in several prostate cancer cell lines
Source: Transl Oncol. 2022 Sep 18;26:101543. doi: 10.1016/j.tranon.2022.101543 (PMC9489499; doi:10.1016/j.tranon.2022.101543)
Supplement: Supplementary file 1 [file mmc1.docx]

# **Supplementary information**


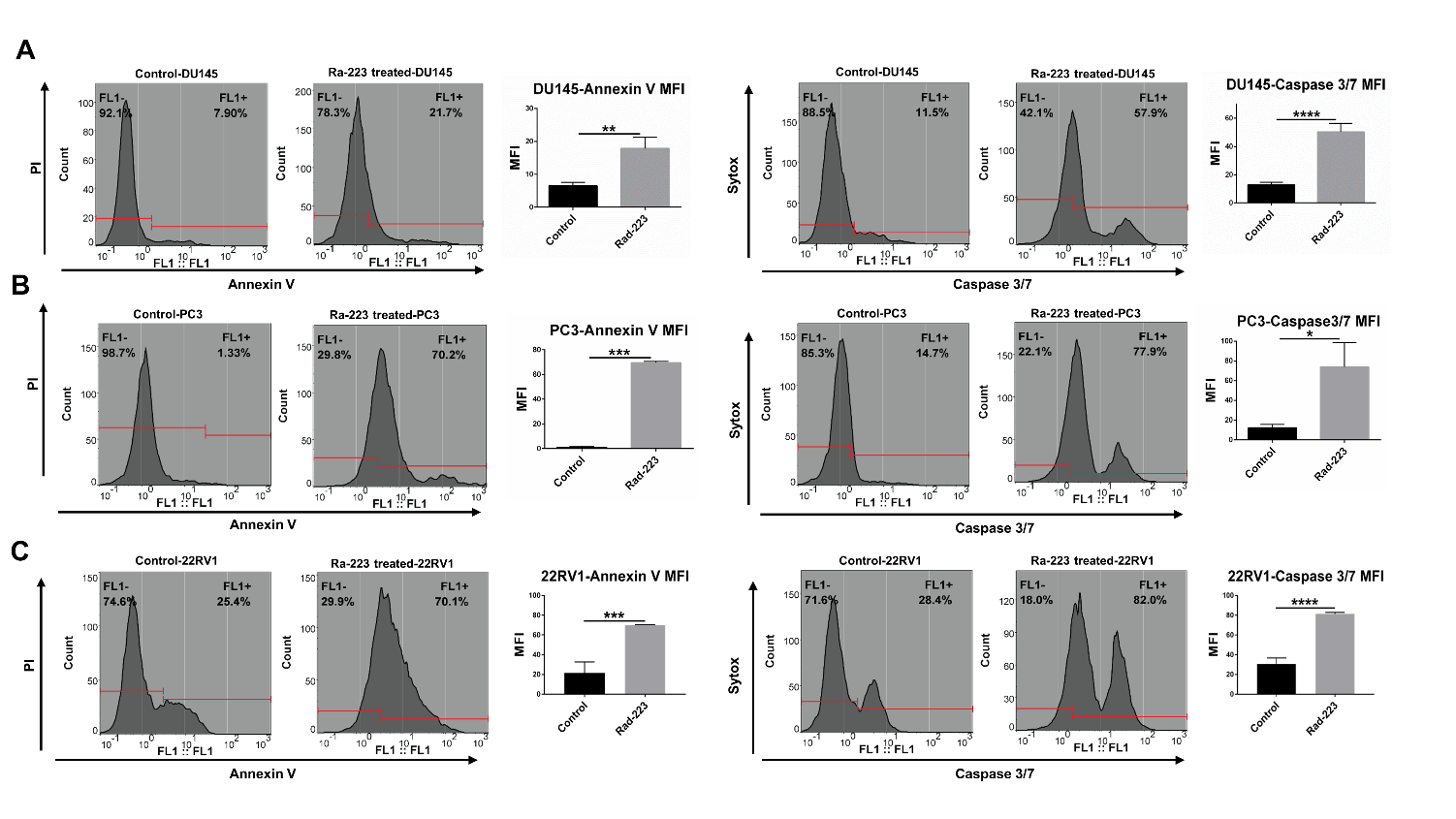


**Supplemental Figure 1.** Annexin V and Caspase 3/7 median fluorescent intensity (MFI) analysis of apoptosis. MFI expression (FITC.A+) of Annexin V and caspase 3/7 of (A) DU145, (B) PC3, and (C) 22RV1 cells after 48 h incubation with 1000 Bq/ml Ra-223 followed by 24h incubation without activity before analysis. Data from at least three independent experiments with mean and SE. Student t-test was used to analyze the statistical differences between samples with * *p*<0.05, ** *p*<0.01, *** *p*<0.001, and **** *p*<0.0001.


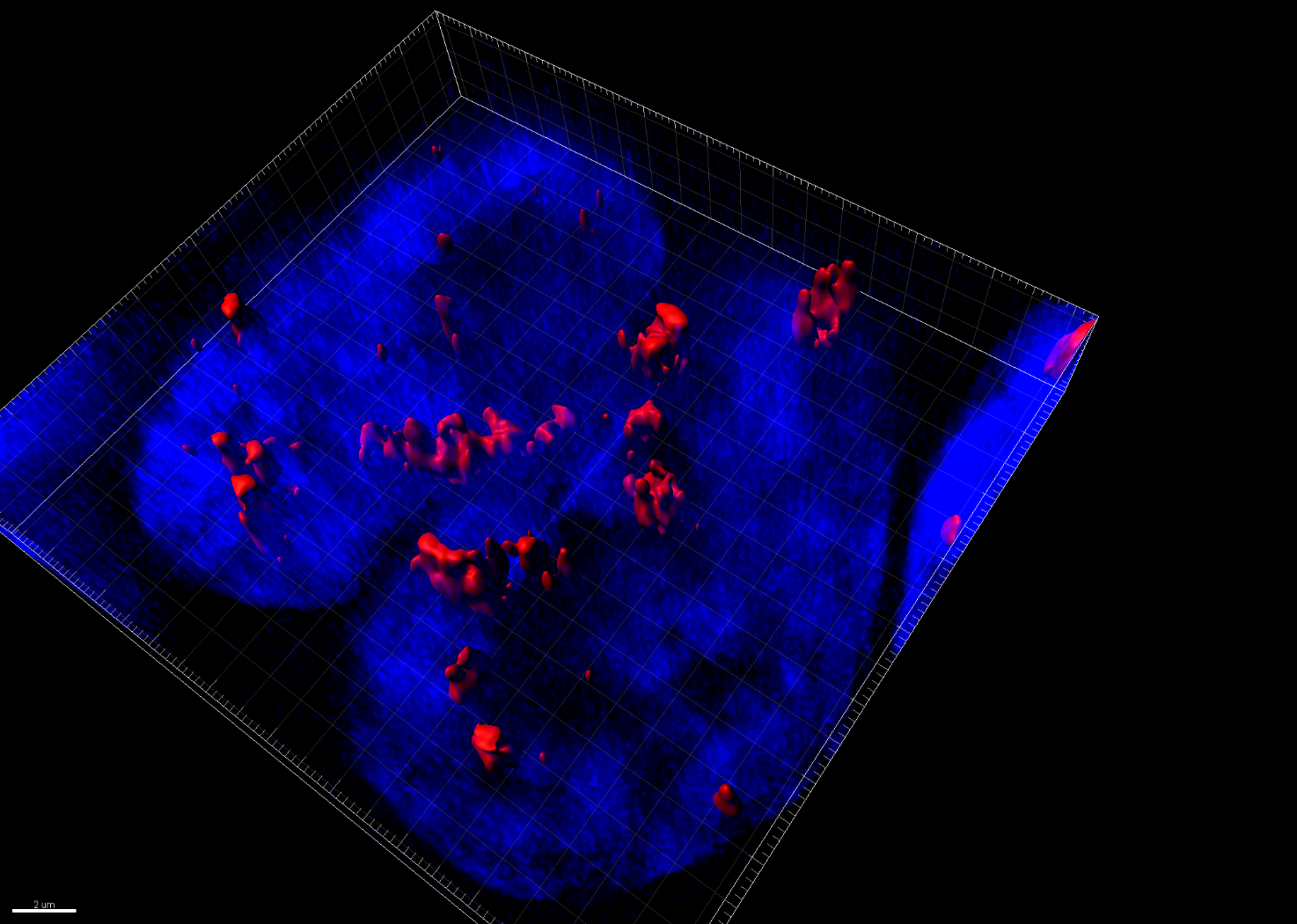
**Supplemental Figure 2.** Ra-223 induces track-like distribution of 53BP1 foci. 22RV1 cells were treated on ice with 100 kBq/ml Ra-223 for 1 h and allowed to repair for 1 h. Cells were stained with the DAPI (blue) and 53BP1 (red) and visualized using the structured-illumination microscopy. The 53BP1 is displayed as surface rendered in Imaris. White bar represents 2 µm.


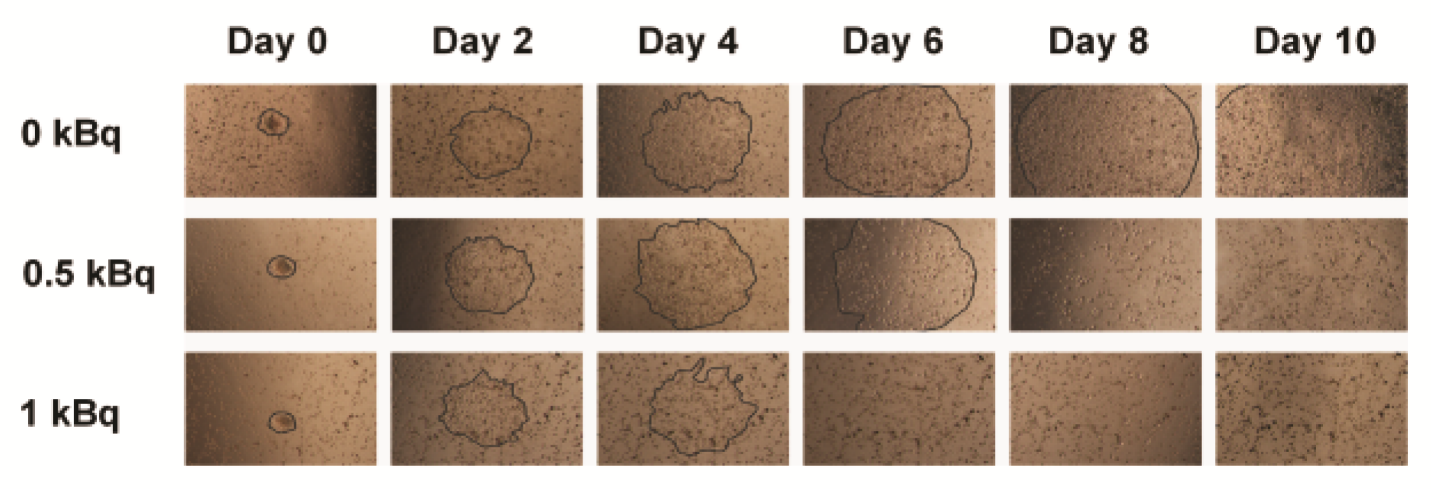


**Supplemental Figure 3.** Ra-223 treated HAp surface affects DU145 cell survival without affecting the cellular outgrowth. DU145 spheroids were plated on HAp surface which was treated with Ra-223 and cellular outgrowth was measured every 2 days. Representative images from 3 independent experiments are shown.


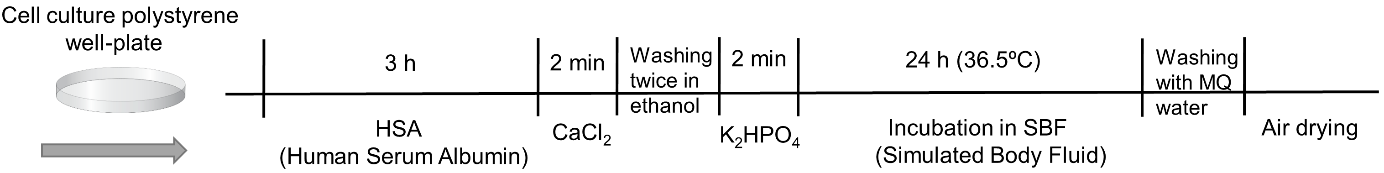


**Supplemental Figure 4.** Schematic workflow of coating cell culture plate with HAp (Adopted with modification from Iijima et al. 2016).

**Supplemental table 1.** Summary of materials, experimental procedure, and exposure time

| Assay type and cell line | Dish type | Treatment  volume (ml) | Activity concentration (Bq/ml) | Exposure time |
| --- | --- | --- | --- | --- |
| Colony formation (free Ra-223)  PC3, 22RV1, DU145 | 6-well plate | 2 | 10, 50, 100, 250, 500 | 8-14 days |
| Colony formation (HAp+Ra-223)  PC3 | 6-well plate | 2 | 500 (2h treatment of HAp) | 8-14 days |
| Spheroid (free Ra-223)  22RV1 | 96-well plate | 0.2 | 100, 250, 500 | 18 days |
| Spheroid outgrowth (HAp+Ra-223)  22RV1,DU145 | 12-well plate (HAp coated) | 2 | 0,500, 1000 (2h treatment) | 10 days |
| DNA repair (flow cytometry)  PC3, 22RV1, DU145 | 6-well plate | 2 | 300,000 | 1 h |
| DSB detection (PFGE)  DU145 | 15ml Falcon tube | 2 | 1,000,000 | 6 h |
| Immunofluorescence foci assay  (PC3, 22RV1, DU145) | 4-well chamber slide | 1 | 100,000 | 1 h |
| Apoptosis  PC3, 22RV1, DU145 | T25 | 2 | 1000 | 48 h |
